# The wide phenotypic spectrum of thiamine metabolism dysfunction syndrome 5 and its treatment

**DOI:** 10.1186/s13023-025-03665-9

**Published:** 2025-04-04

**Authors:** Alice Dallan, Giuseppe Reynolds, Carlotta Canavese, Diana Carli, Maria Luca, Andrea Gazzin, Marco Spada, Francesco Porta, Alessandro Mussa

**Affiliations:** 1 Postgraduate School of Pediatrics, University of Torino, Torino, Italy; 2 Department of Public Health and Pediatric Sciences, School of Medicine, University of Torino, Torino, Italy; 3 Child Neurology Unit, Regina Margherita Children’s Hospital, Torino, Italy; 4 Department of Medical Sciences, University of Torino, Torino, Italy; 5 Pediatric Clinical Genetics Unit, Regina Margherita Children’s Hospital, Torino, Italy; 6 Pediatric Inborn Errors of Metabolism, Department of Pediatrics, Regina Margherita Children’s Hospital, Torino, Italy; 7 Department of Public Health and Pediatrics, Regina Margherita Children Hospital, University of Torino, Piazza Polonia 94, 10126 Torino, Italy

**Keywords:** *TPK1*, Thiamine metabolism dysfunction syndrome 5, Thiamine, Recurring ataxia, Leigh syndrome

## Abstract

Thiamine metabolism dysfunction syndrome 5 (TMDS5) is a rare inborn error of metabolism caused by variants in *TPK1*, leading to reduced TPK levels. This enzyme is crucial for the production of thiamine pyrophosphate, the active form of thiamine, a vital coenzyme in numerous metabolic pathways. The clinical presentation exhibits a diverse range of manifestations. In this review, we explore reported cases in the literature and present two cases representing the extremes of the clinical spectrum: recurrent ataxia and Leigh syndrome. The former phenotype follows a milder course. The second one is characterized by early onset and severe symptoms, including dystonia, epilepsy, and developmental regression, progressing rapidly to severe disability with high mortality. Typically, children exposed to infectious or traumatic triggers display episodes marked by ataxia and dystonia, with periods of good health or only mild disabilities in between. Treatment with the phosphorylated thiamine active bioform, TPP, is more effective in the recurrent ataxia form, especially when initiated promptly at symptom onset. Further studies are needed to identify available biomarkers and establish correlations between different variants, severity, and treatment response.

## Introduction

**Image 1:**
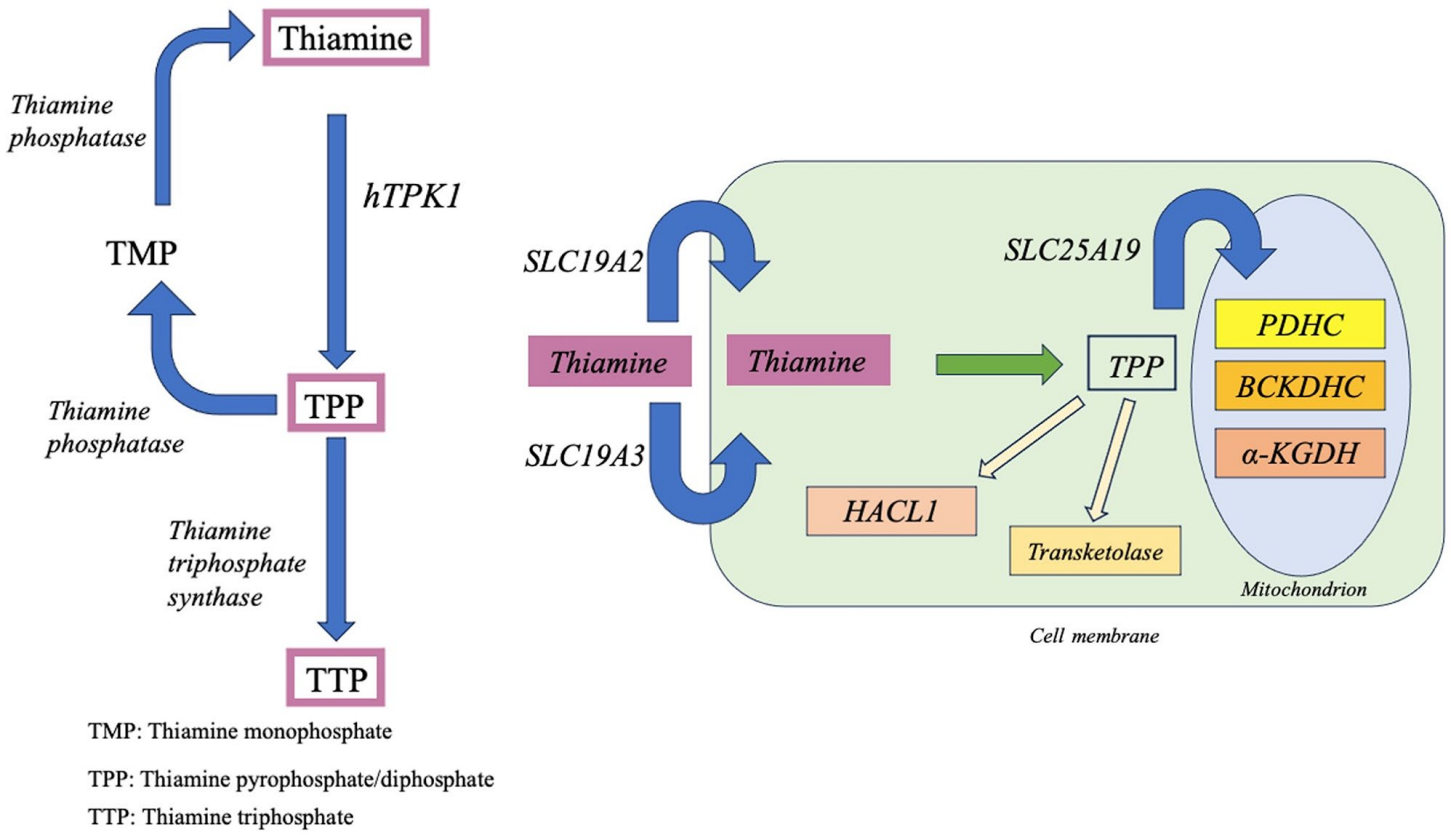
Thiamine pathways: (**A**) Thiamine undergoes phosphorylation to produce thiamine pyrophosphate (TPP) through the action of thiamine pyrophosphokinase 1 (hTPK1). Further phosphorylation by mitochondrial thiamine triphosphosphate synthase results in thiamine triphosphate (TTP). Thiamine can be dephosphorylated to thiamine monophosphate (TMP) and thiamine by thiamine phosphatase. (**B**) The absorption of thiamine in the small intestine and its entry into cells involve transporters encoded by SLC19A2/SLC19A3. Inside cells, thiamine is pyrophosphorylated to its active form (TPP) by hTPK1. TPP serves as a cofactor for three dehydrogenases in mitochondria: (1) pyruvate dehydrogenase complex (PDHC), (2) branched-chain alfa-keto acid dehydrogenase (BCKDH), and (3) alpha-ketoglutarate dehydrogenase (α-KGDH). Outside of mitochondria, TPP functions as a cofactor for Transketolase and 2-hydroxyacyl-CoA Lyase 1 (HACL1)

Thiamine monophosphate, or vitamin B1, is a water-soluble vitamin, mainly absorbed in the small intestine, transported into tissues and cells, and then converted into thiamine pyrophosphate (TPP) by thiamine pyrophosphokinase (TPK) in the cytosol [1]. TPP is the biologically active form of thiamine and serves as an essential coenzyme for the active aldehyde transfers in oxidative decarboxylation of α-keto acids and the transketolase reaction, being therefore a key player in energy metabolism and in multiple metabolic processes in the cytosol, mitochondria, and peroxisome [2]. Thiamine pathways are synthesized in Image 1. Defects in genes encoding proteins with relevant functions in thiamine transport or metabolism cause inborn errors of metabolism and lead to thiamine metabolism dysfunction syndromes. These conditions present clinically with neurological disorders such as recurrent ataxia, dyskinesia, and neurologic development impairment. Currently, five syndromes resulting from errors in this molecular pathway are recognized, attributable to pathogenetic variants in *SLC19A2*, *SLC19A3*, *SLC25A19* and *TPK1* [1, 3].

Thiamine metabolism dysfunction syndrome 5 (TMDS5) is a rare autosomal recessive disorder, caused by variants of *TPK1*, located at 7q35, resulting in decreased TPK levels [4]. This condition causes early childhood onset acute encephalopathic episodes, characterized by a wide spectrum of severity and increased serum and cerebro-spinal fluid (CSF) levels of lactate, usually triggered infections and leading to metabolic decompensation. Its remitting and progressive course leads to neurological dysfunctions and finally results in ataxia, dystonia and loss of motor skills [5]. Less frequently, cases with a very early and more severe clinical presentation, resembling Leigh’s disease, have been described [6].

In this report, we describe two patients with *TPK1* deficiency, presenting with significantly distinct clinical features, highlighting the extensive variability within the disease spectrum.

## Case presentation 1

Patient 1 was a seven-year old girl presenting at the emergency department with acute episode of encephalopathy with dramatic ataxia and hypotonia, worsening along the last 10 days: she complained loss of equilibrium, retropulsion evident even in the sitting position, inability to stand without support, difficulty in swallowing.

Her recent history was silent for infection or any episode of fever and negative for immunizations. The girl was the first child of a couple of healthy non-consanguineous Caucasian parents. The father had a daughter from a previous relationship, who died at one year of age because of spinal muscular atrophy.

The patient had a regular neuropsychological development up to age of 4 when she presented with a first episode of acute encephalopathy and ataxia during influenza infection with fever. In that occasion, the diagnostic workup showed negative results in all biochemical and cultural tests performed, as the instrumental diagnostics, except for an increased blood lactates (3.4 mg/l) and positivity to influenza A virus on a nasal swab. EEG showed significant slowing of the background activity with some paroxysmal discharges. The patient’s clinical conditions progressively improved with steroid (desametasone) and antiviral treatment and she was discharged after 20 days with a diagnosis of encephalitis/cerebellitis due to influenza A virus. She had an incomplete recovery persisting slight motor clumsiness, stable at follow-up controls; cognitive evaluation (WPPSI-III scale) described a total QI of 86 with mild learning difficulties at school.

At the second hospital admission, the patient presented dysarthria, dysphagia, generalized hypotonia with inability to stand alone, even in sitting position, and unsteady wide-base gait, allowed only if supported. A horizontal bilateral nystagmus was observed. The strength-resistance test at the limbs showed rapid exhaustion; cerebellar tests were positive for dysfunction. The remaining physical examination was normal and vital parameters normal. In the setting of emergency department blood and urine test were performed, and sampling of cerebrospinal fluid collected to perform chemical-physical examination, cultural tests and neurotropic virus search, all were resulting normal. Antibody tests for autoimmune encephalitis were negative. An EEG detected diffuse slowing with paroxysmal discharges, and the brain MRI with spectroscopy showed no relevant findings. The SARS-CoV2 PCR test was negative, with the serological test suggesting a previous infection (IgG SARS-CoV2 54 U/ml). Intravenous Ig 2 g/kg treatment was administered, in the suspect of post-SARS-CoV2 infection encephalopathy, with slow and only partial benefit.

Metabolic and toxicologic screening were normal. A genetic test aimed at identifying variants in genes associated with neurodevelopment or movement disorders was performed by Next Generation Sequencing (NGS) gene panel retrieved two variants in the *TPK1* gene (NM_022445.4):*TPK1* c.501 + 4 A > T, classified as pathogenetic according to American College of Medical Genetics (ACMG) criteria, and *TPK1*:c.152G > T; p.Arg51Leu, classified as a variant of uncertain significance (VUS) according to ACMG criteria [7]. The first variant, *TPK1*:c.501 + 4 A > T, caused the substitution of a nucleotide, fully conserved in vertebrates, within the splicing consensus region, predicted to cause aberrant splicing by all in silico tools. The variant was already reported in compound heterozygous state in 3 different individuals with TMDS5 [4, 8], one of which had a phenotype extremely similar to our patient. Experimental studies showed that this splice-site *TPK1*:c.501 + 4 A > T variant decreases the splice efficiency of exon 7 [4]. The variant is reported in ClinVar (variation ID 215275). Given these considerations, the variant *TPK1*:c.501 + 4 A > T was classified as pathogenetic. The second variant *TPK1*:c.152G > T; p.Arg51Leu, was not previously reported in literature or database for human disease variants. The variant was predicted to be tolerated by in silico tools. Functional data supported pathogenicity, because missense variants in *TPK1* are typically associated with disease. Furthermore, a different missense variant in adjacent codon, *TPK1*:c.148 A > C, p.(Asn50His), has been reported in a compound heterozygous state with the variant *TPK1* c.501 + 4 A > T in 2 patients [4], suggesting that this region of the protein is functionally important. The two variants were confirmed by Sanger sequencing and verified as in trans as inherited the first form the mother, the second from the father. The *TPK1*: c.152G > T;p.Arg51Leu variant is classified as likely pathogenic according to ACMG criteria based on low frequency in population databases (GnomAD frequency ƒ = 0.00000399) and the application of PM3 ACMG criteria for variant classification [7]. Thiamine oral supplementation (300 mg/die) has been commenced. On this treatment, di-phosphorylated thiamine (B1-TPP) in a blood sample was in the normal range (29 µg/l - reference 28–85 µg/l). Metabolic tests showed normal lactate and α-ketoglutaric acid levels in urine.

Neurologic and logopedic evaluations were run daily and showed progressive improvement both in ataxia and dysarthria, with reinstatement of sitting position first and erected position then, until walking with help. The dysphagia also improved, allowing soft feeding. The patient was finally discharged after 1 month, carrying on neurologic and logopedic follow-up and continuing substitutive therapy with thiamine. At the last follow-up evaluation (after 15 months) she showed general improvement in clinical conditions: she was able to walk unattended for short distances, despite persisting ataxia, dysarthria and slight hypotonia. No dysphagic symptoms were detected and she attended school with support teacher.

## Case presentation 2

**Image 2:**
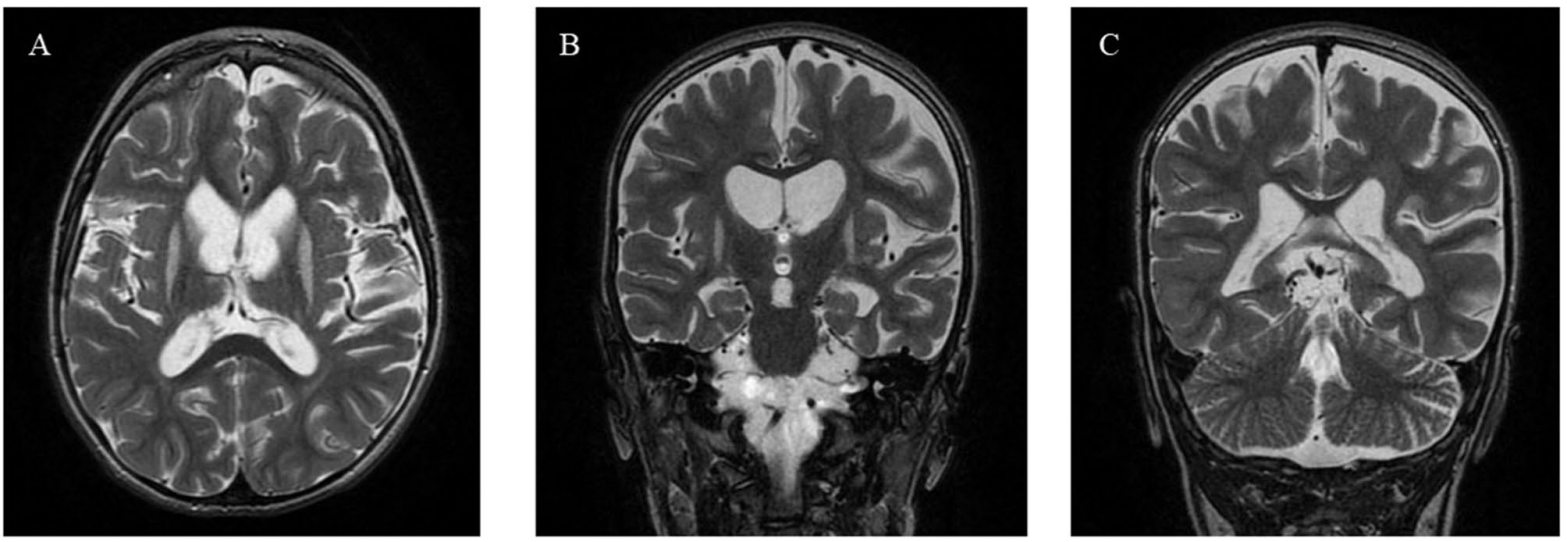
Magnetic Resonance Imaging (MRI) performed at 4 years of age in Patient 2 revealing parenchymal depletion with enlargement of subarachnoid spaces (**A**), alterations in the putamen, striatum, and caudate nuclei (**B**, **C**)

Patient 2 is a second-born male child from consanguineous parents (first cousins) of Moroccan origin. The older brother and parents are in good health, with no reported family health issues. He was born at term after an uneventful pregnancy, with normal intrauterine development and initial adaptation. At 5 months of age, he was admitted to a primary-level hospital for the first time due to weight loss and decreased urine output, in the absence of fever. He was diagnosed and treated for a urinary tract infection and subsequently discharged. At 6 months of age, he was readmitted for developmental regression and trunk and limb hypotonia, leading to referral to the metabolic diseases center. During clinical evaluation, he presented as irritable, with very poor motor skills and dyskinetic movements. He made attempts at head control and had some discrete eye tracking, with substantial delay in motor skills achievements for its age. The rest of the physical examination was unremarkable. Blood chemistry tests showed mild subclinical hypothyroidism. Brain magnetic resonance imaging was normal, except for an arachnoid cyst outside the brain with no clinical correlation. Metabolic tests including plasmatic and urinary amino acids, urinary organic acids, pipecolic acid, emogasanalysis with lactate and glycemia, array CGH and FMR1 region repeats were normal. A trio-based whole exome sequencing revealed the homozygous variant *TPK1*: (NM_022445.4): c.664G > C p.(Asp222His)in exon 9, inherited from the heterozygous parents. The variant was classified as pathogenetic given the extremely low frequency in GnomAD population databases, functional data and in silico predictions. Moreover, the variant was already reported in literature in another children with a severe TMDS5 phenotype [9]. Therefore, therapy with thiamine 300 mg/day was initiated, resulting in improvement in head control and hypotonia and reduced irritability. Subsequently, the child was lost to follow-up but continued physiotherapy and treatment with thiamine for 3 years. At 4 years and 5 months, he was readmitted to the hospital due to deteriorating general condition. He presented severely suffering and cachectic, with muscle atrophy in a malnutrition setting. Clonazepam was prescribed for agitation. Hypotonia was pronounced, with complete inability to control the trunk and head and to walk. Neurodevelopmental delay was severe, characterized by an inability to speak and to engage with stimula from the external world. His head circumference was below the third percentile. He had laryngomalacia and dysphagia, which required discontinuation of oral feeding and placement of a percutaneous endoscopic gastrostomy tube. A new MRI showed symmetric bilateral signal alterations selectively involving the striatum, which appeared atrophic, with predominant changes in the putamen and caudate nuclei, and parenchymal depletion with enlargement of subarachnoid spaces (Image 2). Currently, the child has a stable condition with improved overall nutritional health thanks to supportive nutrition. He continues thiamine therapy at 300 mg/day (30 mg/kg), which was later supplemented with biotin, but has not shown clinical neurological improvement.

## Discussion

TMDS 5 is the last entity recognized as part of the spectrum of genetic defects of thiamine transport and metabolism. It should be considered in newborns, toddlers and children with symptoms like ataxia and dystonia as well as lactate elevation during metabolic crises. Clinical, radiological and biochemical diagnostic criteria are stated and include acute or recurrent episodes of encephalopathy, with predominant but not exclusive motor involvement, normal total thiamine blood levels, low TPP in blood, muscle and/or fibroblasts, high excretion of alpha-ketoglutaric acid in urine, MRI abnormalities (hyperintensity in basal ganglia and cerebellum), trigger events such as infections, increases lactate in blood and/or CSF. As demonstrated in our two case reports, highlighting the two ends of the clinical spectrum, very different clinical presentations are possible: recurrent ataxia and Leigh syndrome ataxia phenotypes. The first one is follows a more indolent course and usually affects children who, following infectious or traumatic triggers, experience episodes chiefly characterized by ataxia and dystonia. In-between such episodes, these children are either healthy or exhibit very mild disabilities. In contrast, Leigh syndrome is characterized by an early onset, severe symptoms such as dystonia, epilepsy, and developmental regression. Rapid progression towards a state of severe disability with high mortality is part of the natural history of this presentation.

TMDS5 was first described in 2011 by Mair et al., who reported five individuals from three families presenting with variable degrees of ataxia, psychomotor retardation and progressive dystonia [4]. Including our 2 patients, 32 cases are now documented in literature, summarized in Table 1. The age of onset in described patients is between 1 month and 7 years. 37.5% of cases presented before the first year of life, and 75% before the age of 3. The female-to-male ratio is 1.3:1. The brain MRI was normal at onset in 7 of them, as it was in our patients. In two patients with severe form, described by Mayr et al., as in our Patient 2, a subsequent MRI showed diffuse degeneration of white matter. Affected individuals had a significant elevation of α-ketoglutaric acid in urine and decrease of thiamine and TPP in blood. The molecular diagnosis of TMDS5 in these patients is largely connected with the implementation of Whole Exome Sequencing or NGS Panels in recent years [10]. This was relevant especially in our cases in which no clue was present at a metabolic or imaging level, hampering a correct clinical framing of the disorder. By NGS analysis we have identified three different mutations in TPK encoding gene, *TPK1* c.664G > C and c.501 + 4 A > T, notoriously pathogenetic, and *TPK1* c.152G > T, p (Arg51Leu), which was a variant with uncertain significance (VUS). As it was not possible to establish whether the variants in Patient 1 were in cis or in-trans, it was necessary to search for the variants on parental DNA. In an emergency setting it is therefore preferable to perform such kind of test directly on a trio-based analysis to reduce turnaround and management times.

**Table 1:** Cases of thiamine metabolism dysfunction syndrome 5 reported in literature

Author	Sex	Variant (mutation)	Infective trigger	Age of onset (y)	Main clinical features	Abnormal MRI	Thiamine treatment	Outcome
Mayr, 2011	F	c.148 A > C (p.Asn50His) c.501 + 4 A > T (Val119_Pro167del)	Yes	1.25	Encephalopathy, developmental delay and regression, hypotonia, lactic acidosis	Normal	No	Death, metabolic crisis
	F	c.148 A > C (p.Asn50His) c.501 + 4 A > T(Val119_Pro167del)	Yes	1.5	Ataxia, dystonia, microcephaly	NA	No	Death, infection
	F ^1^	c.119T > C (p.Leu40Pro)	Yes	4	Seizure, dystonia, rigidity, dysarthria	Normal at first, then global atrophy with increased globus pallidus signal intensity	Yes	Invalid
	M ^1^	c.119T > C (p.Leu40Pro)	Yes	4	Dystonia, microcephaly, loss of speech	Normal	Yes	Improved
	M	c.179_182delGAGA (p.Arg60LysfsX52) c.656 A > G (p.Asn219Ser)	Yes	2	Ataxia, dysarthria, seizures	Normal at first, then changes in the white matter of the cerebellum, increased signal intensities in corticospinal tract at the medulla oblongata, and slightly increased intensities in the dorsal pons.	Yes	Improved
Banka, 2014	M	c.479 C > T (p.Ser160Leu)	Yes	2.5	Encephalopathy, ataxia, dystonia, quadriparesis, developmental delay, dysphagia, dysarthria	High signal in dentate nucleus	Yes	Improved
	F	c.664G > C (p.Asp222His)	No	0.67	Dystonia, hypotonia, dysphagia, developmental delay	Delayed myelination and altered density of basal ganglia	Yes	Invalid
Fraser, 2014	F ^1^	c.604T > G (p.Trp202Gly)	Yes	0.58	Encephalopathy, seizure, hypotonia, lactic acidosis, dysarthria, respiratory failure, developmental delay and regression	Abnormal T2 signal in basal ganglia and thalamus	No	Death, metabolic crisis
	M ^1^	c.604T > G (p.Trp202Gly)	Yes	0.33	Hypotonia, facial palsy, dysarthria, developmental delay	Abnormal T2 signal in basal ganglia and thalamus	Yes	Improved
Invernizzi, 2017	F	c.656 A > G (p.Asn219Ser)	Yes	1.75	Encephalopathy, ataxia, dystonia	Abnormal signal in bilateral putamen dentate nucleus	Yes	Improved
Ortigoza-Escobar, 2017	NA	c.365T > C (p.Ile122Thr)	No	0.08	Seizure, hypotonia, hepatopathy	Abnormal signal in putamen, globus pallidus, dentate nucleus of cerebellum	No	Death
Mahajan, 2017	M ^1^	c.119T > C (p.Leu40Pro)	No	4.67	Dystonia	Abnormal basal ganglia signal	Yes	Invalid
	F ^1^	c.119T > C (p.Leu40Pro)	No	NA	Dystonia	NA	Yes	Invalid
Huang, 2019	F ^1^	c.83 A > G (p.Leu28Ser)	Yes	0.08	Encephalopathy, seizure, ataxia, dystonia	Abnormal symmetrical signal of the cerebellar dorsal medulla and dentate	Yes	Improved
	M ^1^	c.83 A > G (p.Leu28Ser) (presumed)	NA	First months	Seizure, ataxia, dystonia	NA	No	Death
	F ^1^	c.83 A > G (p.Leu28Ser) (presumed)	NA	First months	Seizure, ataxia, dystonia	NA	No	Death
Lina Zhu, 2019	M	c.263G > A (p.Cys88Tyr) c.226 A > G (p.Arg76Gly)	Yes	0.33	Encephalopathy, seizure, ataxia, dysarthria, dystonia	Long T1 and T2 abnormal signals appeared in the hippocampus of bilateral basal ganglia, DWI showed obvious signals, and the subarachnoid space of bilateral frontotemporal lobe was widened	Yes	Invalid
Nyhan, 2019	F	c.311delG (p.104SerfsX9) c.426G > C (p.Leu142Phe)	Yes	2.5	Ataxia, dystonia, nystagmus, optic atrophy, dyssomnia, deafness	Bilateral striatal symmetrical T2 hyperintensity, cerebellar and brainstem volume reduction	Yes	Invalid
Bizhen Zhu, 2020	M ^1^	c.382 C > T (p.Leu128Phe)	Yes	3.5	Ataxia, dysarthria	Immature cerebellar vermis	Yes	Improved
	M ^1^	c.382 C > T (p.Leu128Phe)	Yes	2	Ataxia, dysarthria, dysphagia, dystonia	Normal	Yes	Improved
Dongxiao Li, 2020	F	c.395T > C (p.Phe132Ser) c.614-1G > A (p.?)	Yes	1	Encephalopathy, ataxia, dystonia, quadriparesis, dysarthria, developmental delay and regression	Abnormal signals in the thalamus and cerebellar hemispheres of the caudate putamen bilaterally, and mild progressive cerebellar atrophy	Yes	Improved
Au, 2020	F ^1^	c.502-1G > T (p.?) c.167 C > T (p.Ser54Leu)	No	3	Encephalopathy, dystonia, intention tremor, lactic acidosis	Normal	Yes	Invalid
	F ^1^	c.502-1G > T (p.?) c.167 C > T (p.Ser54Leu)	No	7	Encephaolopathy, dystonia, seizure	Normal	NA	NA
Jue Wang, 2021	F	c.161 C > T(p.Ser54Leu)	Yes	2.67	Encephalopathy, seizure, ataxia, dystonia, dysarthria, dysphagia, respiratory failure	Symmetrical patchy long T1 and T2 signals of bilateral cerebellar hemispheres and splenium of the corpus callosum	No	Death, respiratory and circulatory failure
Rusch, 2021	F	c.501 + 4 A > T (p.Val119_Pro167del) c.576T > G (p.Cys192Trp)	Yes	0.5	Encephalopathy, ataxia, dystonia, hypotonia, developmental delay and regression, loss of speech	Bilateral basal ganglia, thalamus, and dentate nucleus had focal t2 hyperintense lesions with limited diffusion	Yes	Improved
Eckenweiler, 2021	NA	c.501 + 4 A > T (p.Val119_Pro167del) c.576T > G (p.Cys192Trp)	Yes	0.92	Encephalopathy, ataxia, dystonia, dysphagia, developmental delay	Symmetrical T2 hyperintensity bilaterally in the posterior putamen and dentate nucleus, punctate diffusion restriction and slight lactate peak in the basal ganglia	Yes	Improved
Shaowei Li, 2021	M	c.382 C > T (p.Leu128Phe)	Yes	1.08	Ataxia	Patchy low-density shadow on the right cerebellum	Yes	Improved
Zhao, 2023	M	c.513delG (p.Arg171Ser fs*3) c.382 C > T (p.Leu128Phe)	Yes	1.67	Ataxia, dysarthria, dystonia	Symmetrical patchy long T2 signals in bilateral basal ganglia and thalamus of cerebellar dentate nucleus	Yes	Improved
	M	c.263G > A (p.Cys88Tyr) c.465_467delAAT (p. Leu156del)	No	0.08	Seizure, dystonia, nystagmus, developmental delay	Abnormal signal foci of bilateral caudate head, lentiform nucleus and dentate nucleus of bilateral cerebellar hemispheres were roughly symmetrical. Both hemispheres are less developed	Yes	Death, respiratory and circulatory failure
Thompson, 2023	F	c.646G > A (p.Ala216 Thr) c.491T > C (p.Leu164Pro)	Yes	2	Encephalopaty, ataxia, dystonia, seizures	T2/Flair signal prolongation within the occipital lobe with white matter tracts with no restricted diffusion or contrast enhancement	Yes	Improved
Our study	F	c.152G > T (p.Arg51Leu) c.501 + 4 A > T	Yes	4	Ataxia, dysarthria, dysphagia	Normal	Yes	Improved
	M	c.664G > C (p.Asp222His/Asp222His)	Doubtful	0.8	Encephalopathy, dystonia, dysphagia developmental delay	Normal at first, then symmetric bilateral signal alterations selectively involving the striatum, which appeared atrophic, with predominant changes in the putamen and caudate nuclei, and parenchymal depletion with enlargement of subarachnoid spaces	Yes	Invalid

^1^ couples of siblings

NA not applicable

According to literature, thiamine supplementation (100–500 mg/day) in TPK-deficient patients has led to clinical improvement and even normal neurodevelopment in early treated patients: we can hypothesize that a large amount of precursor may increase the amount of di-phosphorylated active form of thiamine in spite of a low enzymatic activity [1, 6, 11]. However, it must be underlined that, given the location of the enzymatic defect (TPK which catalyzes the conversion of thiamine to TPP, the active form), a more effective therapy could probably be represented by supplementation with TPP rather than thiamine. Unfortunately, TPP is not available as a drug in Italy. Seven of the patients (27% of treated individuals) did not respond to thiamine treatment, and this could be explained by a very low level of residual enzymatic activity [9, 12]– [15]. Likely, the diphosphorylated form of biotin would better fit as a treatment, although it is not commercially available. One of the patients died during metabolic crises despite treatment [6]. Six patients (18.75%) died before accessing the therapy [4, 16]– [19]. We administered high dose thiamine (300 mg/die) in both our patients with improvement of ataxia, dysmetria and dysphagia at short-term follow up (3 month). The subsequent treatment response demonstrates significant differences: Patient 1, currently at 15 months of follow-up from diagnosis, maintains good overall conditions and has gradually regained the ability to walk, albeit with ataxic features and with support for long distances. Patient 2, on the other hand, after an initial response to treatment, has progressed towards severe disability. As demonstrated by two previous case reports, variant c.501 + 4 A > T found in Patient 1 is associated with a better response to therapy [2, 8]. Up to now, follow-up studies are very limited, and it is still unknown if neurological abnormalities may even develop after long-term follow-up. The low number of known patients in the literature and the numerous pathogenic variants described do not allow for making statistical associations between genotype and phenotype or between genotype and treatment response.

## Conclusions

TMDS5 is a rare disorder of inborn error of metabolism caused by *TPK1* pathogenic variants. Genetic defects of thiamine transport and metabolism should be considered in all patients with episodes of recurrent encephalopathy and/or dystonia as well as lactate elevation during metabolic crises. Clinical, biochemical and radiologic criteria are helpful to make the diagnosis, but such rare disorders may have heterogenous and non-specific presentation, often hampering a rapid and correct clinical framing of the condition. For this reason, rapid genetic testing analysis by NGS plays a crucial role in the differential diagnosis of children with ataxia/motor regression. Thiamine and TPP measurement in both plasma and CSF, as well as an estimate of TPK residual are likely diagnostic, but such tests are mostly unavailable even in laboratories specialized in the diagnosis of inborn error of metabolism. Parallelly, treatment with the phosphorylated thiamine active bioform TPP would likely be the best treatment approach, but it is not available in many countries. In the absence of quickly available biomarkers, a trial treatment with high doses of thiamine should therefore be considered, even before results of whole exome sequencing are available. Further studies are needed to assess all the features of the TMDS spectrum, which ends are represented by the two clinical phenotypes described in this report, Leigh-like vs. non-Leigh-like, as well as correlation between the different variants, severity, and response to treatment.

## Data Availability

The dataset supporting the conclusions of this article is included within the article.
